# Lung cancer—a one-way ticket

**DOI:** 10.1093/jncics/pkae098

**Published:** 2024-11-04

**Authors:** Drew Moghanaki, Michelle Ann Eala, Jill Feldman, Terri Ann DiJulio, Peter Gorayski

**Affiliations:** Department of Radiation Oncology, University of California, Los Angeles, Los Angeles, CA, United States; Department of Radiation Oncology, University of California, Los Angeles, Los Angeles, CA, United States; Patient Representatives, United States; Patient Representatives, United States; Department of Radiation Oncology, Royal Adelaide Hospital, Adelaide, Australia

*The physician’s duty is not to stave off death or return patients to their old lives, but to take into our arms a patient and family whose lives have disintegrated and work until they can stand back up and face—and make sense of—their own existence.*

*     *—Paul Kalanithi, MD

## A journey beyond the clinical diagnosis

For individuals diagnosed with lung cancer, their journey often feels like being handed a “one-way ticket” to a destination unplanned—a voyage where life as they knew it may never return to its original state. Even in the face of a cure, their experience is marked not only by the clinical challenges of a complex illness but also by psychosocial, social, and financial burdens that are profound and enduring, extending to their families, friends, and caregivers. This journey is also affected by the stigma that lung cancer carries, shrouded in nihilism and fear, which remains a heavy cloak even after the disease may be no longer detectable. Despite unparalleled progress in medical science offering longer, healthier lives for those affected, the ever-present shadow of mortality statistics profoundly shapes the patient’s journey for the rest of their lives, underscoring that the path for individuals with lung cancer can remain irrevocably altered. Our coauthors, consisting of 2 patients with lung cancer and 3 radiation oncologists who treat lung cancer, collaborated on this narrative to convey the complexity of challenges faced by patients with lung cancer. By shedding light on these challenges, many of which are rarely discussed in the clinic, we hope that providers will consider a more holistic approach to care that reaches beyond the confines of the clinical setting. Our ultimate goal is to transform this seemingly one-way journey for patients into a pathway more filled with hope, support, and comprehensive care. 

## Navigating the maze of cancer care

The path for patients with lung cancer is fraught with fear and confusion, exacerbated by medical jargon, complex treatment pathways, and fragmented health-care delivery systems that are disruptive and burdensome. Patients face new realities in every aspect of life while confronted with making difficult treatment decisions; they know there is no room for error while struggling to understand their options and weighing the benefits of treatment against the potential side effects. Amid anxiety and distress, clear thinking becomes difficult, leaving patients to manage their fears, do their own research, and seek care from an array of specialists generally in a hurried setting. The varying advice from different specialists has the potential to sow doubt and mistrust, further challenging patients’ faith in their care regimen.

## Social factors affecting cancer care

The social factors affecting lung cancer care are often overlooked, such as taking time off work, managing childcare or elder care, and possibly needing temporary accommodation for and/or transportation to distant treatment facilities. These non-medical social factors heighten financial toxicity, significantly affecting diagnosis, treatment, outcome, and quality of life, even after treatment completion. For example, it has been shown that patients with lung cancer who experience a greater travel burden to a cancer care facility have significantly lower overall survival (1).

## Battling the stigma of a diagnosis

The stigma associated with lung cancer, often tied to smoking and shame, adds psychosocial and emotional burdens to patients and families. Smoking is an addiction, while other risk factors are often ignored. This stigma overlooks the multifactorial nature of lung cancer, where environmental and genetic factors also play significant roles beyond cigarette use. The undue focus on smoking history detracts from the empathetic care and support these patients deserve. The psychological impact of a lung cancer diagnosis is significant—a study of more than 10 000 patients with cancer revealed that patients with lung cancer have higher levels of anxiety and depression at the time of diagnosis compared with other cancer sites (2).

## Women and lung cancer

The challenges of lung cancer care may be magnified in women with social factors that may be more significant than appreciated. Women often have competing responsibilities with homemaking, childcare, and work, leaving them with limited time and resources to care for themselves in the face of a lung cancer diagnosis. Much of unpaid cancer caregiving is delivered by women (3). The stigma associated with lung cancer may be misplaced in some patients; more than 50% of women with lung cancer worldwide do not smoke (4). Risk exposures outside of tobacco use are higher in women, including secondhand smoke, household solid fuel, air pollution, and biomass exposure from burning coal and wood for cooking in unventilated stoves (5). Women are also underrepresented in lung cancer trials such as screening (16%) and therapies (39%) (3); consequently, gaps remain in terms of drawing meaningful conclusions that can be specifically applied to women with lung cancer. It is, therefore, unsurprising that emotional distress is highest among women with lung cancer when compared with other cancer types (2).

## Advancements in care: a beacon of hope amid fear

There have been undeniable advancements in the treatment and management of lung cancer. Innovations in early detection, targeted therapies, and a growing appreciation for supportive care has significantly improved survival and quality of life. However, living with profound uncertainty, fear of the unknown, recurrence, and potential metastases looms large, casting a shadow over these advancements. Balancing the optimism brought by these medical strides with ongoing emotional and psychological hurdles is challenging for patients and families.

## The anxiety of surveillance

Post-treatment surveillance, rather than offering relief, often exacerbates anxiety, resulting in a phenomenon known as “scanxiety” (6). The understanding that metastatic lung cancer is incurable and that even early-stage disease might recur fosters a continuous state of uncertainty. This awareness underscores the urgent need for a comprehensive approach to care that addresses patients’ physical, emotional, and psychological well-being. Additionally, caregivers must not be overlooked during this phase of cancer care, because lung cancer caregivers have higher rates of anxiety and depression at 6 and 12 months post-diagnosis compared with other cancer caregivers (7).

## A call to arms for clinicians

As clinicians, we find ourselves in a privileged position to affect meaningful change in our patients’ lung cancer journey. Our paths through medical training are often focused on applying medical science and hard data to patient care, which can sometimes overlook the human experience of illness beyond the healthcare environment. We manage highly complex multidisciplinary care and have an incredible opportunity to make a profound impact on the lives of patients and families with the diagnostic and treatment options available. Through direct patient encounters we begin to see the world through their eyes, recognizing the vast scope of support we can offer beyond traditional treatments (2). Yet, we can do even better by embracing the art of oncology, and remembering the timeless wisdom of Hippocrates, as quoted by Lynn Schuchter: “To cure sometimes, to relieve often, and to comfort always.”

To truly support our patients, we must simplify medical messaging to make complex information accessible. We must prioritize emotional well-being and engage in empathetic and compassionate listening to validate patient experiences beyond the symptoms we ask about. We have a responsibility to address and dispel the stigmas surrounding lung cancer, educating patients and the broader community so they better understand the complexities of a smoking addiction and other, yet to be identified, causes for lung cancer beyond cigarettes.

We must also help identify resources to support patient navigation, referrals to support groups, and engagement with advocacy organizations to help patients gain access to a network of care that extends beyond immediate care providers. By expanding our patients support network, they will be better able to navigate their diagnosis and have a greater understanding with less isolation.

Our purpose here is to create a call to action for all clinicians to broaden our gaze, to remember the human stories behind the immediate clinical challenges, and to address the profound emotional and psychosocial aspects that lung cancer has on each of our patients’ lives. It is a call to recognize and act on the outlined needs above to ensure our patients do not face their journies alone. More importantly, it serves as a reminder that although extending survival is important, it is not the only important endpoint. Patients do not just want to survive—they want to live and enjoy their lives while they are surviving. Reflecting on the roles of lung cancer care providers and caregivers, we must consider how we can shift our perspective to consistently and clearly see the broader effects of lung cancer at all times. In doing so, we can transform a lung cancer diagnosis from a one-way ticket into a journey of hope, support, and comprehensive care.
